# Health impact and cost-effectiveness of a private sector bed net distribution: experimental evidence from Zambia

**DOI:** 10.1186/1475-2875-12-102

**Published:** 2013-03-18

**Authors:** Richard Sedlmayr, Günther Fink, John M Miller, Duncan Earle, Richard W Steketee

**Affiliations:** 1, 1410 Broadway, New York, NY, 10018, USA; 2 Harvard School of Public Health, 677 Huntington Avenue, Boston, MA, 02115-6018, USA; 3 PATH Malaria Control and Evaluation Partnership in Africa, Postal Net Box 370, Private Bag E-10, Lusaka, Zambia

**Keywords:** Public-private partnership, Zambia, Cost-effectiveness, Insecticide-treated nets

## Abstract

**Background:**

Relatively few programmes have attempted to actively engage the private sector in national malaria control efforts. This paper evaluates the health impact of a large-scale distribution of insecticide-treated nets (ITNs) conducted in partnership with a Zambian agribusiness, and its cost-effectiveness from the perspective of the National Malaria Control Programme (NMCP).

**Methods:**

The study was designed as a cluster-randomized controlled trial. A list of 81,597 cotton farmers was obtained from Dunavant, a contract farming company in Zambia’s cotton sector, in December 2010. 39,963 (49%) were randomly selected to obtain one ITN each. Follow-up interviews were conducted with 438 farmers in the treatment and 458 farmers in the control group in June and July 2011. Treatment and control households were compared with respect to bed net ownership, bed net usage, self-reported fever, and self-reported confirmed malaria. Cost data was collected throughout the programme.

**Results:**

The distribution effectively reached target beneficiaries, with approximately 95% of households in the treatment group reporting that they had received an ITN through the programme. The average increase in the fraction of household members sleeping under an ITN the night prior to the interview was 14.6 percentage points (p-value <0.001). Treatment was associated with a 42 percent reduction in the odds of self-reported fever (p-value <0.001) and with a 49 percent reduction in the odds of self-reported malaria (p-value 0.002). This was accomplished at a cost of approximately five US$ per ITN to Zambia’s NMCP.

**Conclusions:**

The results illustrate that existing private sector networks can efficiently control malaria in remote rural regions. The intra-household allocation of ITNs distributed through this channel was comparable to that of ITNs received from other sources, and the health impact remained substantial.

## Background

Despite massive international efforts, malaria continues to be one of the principal causes of ill health as well as mortality in sub-Saharan Africa today with approximately one million deaths per year globally [1,2]. On average, individuals living in highly endemic areas are estimated to suffer at least one bout of malaria per year, resulting in an estimated total number of 225 million malaria cases causing an average of five work days lost [3] and an average direct cost of three to seven US$ for treatment alone [4].

Insecticide-treated bed nets (ITNs) are considered one of the most effective interventions against malaria [5,6] and have been endorsed by the World Health Organization for the global anti-malaria efforts. Comprehensive coverage with ITNs has been shown to lead to a 50 percent reduction in the incidence of uncomplicated *Plasmodium falciparum* malaria episodes in areas with stable malaria, and a reduction of 62 percent in areas with unstable malaria [7].

Over the past decade, many countries in Africa have bolstered efforts to make ITNs widely available through a variety of distribution efforts to scale malaria control interventions [8,9]. These efforts were ushered in through commitments by national political leadership as well as a variety of partners such as the Global Fund and the President’s Malaria Initiative. Among malaria control programmes in Africa, the Zambian National Malaria Control Programme (NMCP) has made notable progress in scaling ITN coverage through rolling mass distributions and targeted distributions to pregnant women and children under age five through antenatal clinics as part of routine care [10]. These efforts have resulted in some of the highest levels of ITN coverage and utilization on the continent [9].

Sustaining high levels of ITN coverage is challenging. Zambia has been particularly successful at increasing the overall levels of coverage through consistent commitments of partners even without much involvement in the private sector for ITN distribution. Traditionally, the private sector in Zambia, and in particular the mining sector, has focused on provision of indoor residual spraying (IRS) services, chemoprophylaxis and treatment services for their employees as part of their strategy to improve the livelihoods of their labour force, and based on the recognition that it may pay to reduce malaria where they operate [11,12]. The challenge in sustaining coverage not only relates to procurement costs, but also to the costs of distributing bulky ITNs to the household level in remote rural areas. As coverage increases, the challenge of tracking those households who have been missed by previous distribution efforts as well as those households needing ITN replacement increases [13]. Engaging additional partners, especially the private sector, in the effort to provide ITNs to those affected by malaria is therefore a priority of Zambia’s NMCP.

In an effort to improve the well-being and productivity of its contract farmers, Dunavant Cotton, an international cotton agribusiness, volunteered to distribute close to 40,000 long-lasting ITNs to randomly selected farmers during the farming season 2010/2011. The ITNs were provided by the Malaria Control and Evaluation Partnership in Africa (MACEPA) project with funding from the Bill and Melinda Gates Foundation through the NMCP. A follow-up survey with participating households was conducted in June and July 2011, which allowed an assessment both of the quality of the distribution and the health impact of the programme.

## Methods

### Study context

Dunavant Cotton Zambia’s core business is the purchase and processing of locally grown cotton, and the marketing of cotton lint. Dunavant is the largest cotton buyer in the country, competing with half a dozen other companies for crop. It signs agreements with smallholder contract farmers at the beginning of the growing season, allowing farmers to obtain farming inputs such as seed and fertilizer on a loan basis in exchange for a commitment to the year’s cotton output exclusively to the Dunavant. The total loan amount is deducted from the delivery at the end of the harvesting season. To guarantee a smooth distribution of resources and a rapid turnaround on cotton purchases, Dunavant maintains a highly decentralized distribution system. In the 2010–11 season, 62 regional warehouses (“sheds”) supervised 1,507 local sales representatives (“distributors”), who coordinate all interactions with locally partnering farmers.

### Study population

Dunavant is active in four of Zambia’s ten provinces: Eastern, Southern, Central and Lusaka. As of 2010, the total population in these provinces is approximately 6.8 million individuals, living in over one million households [14]. As of December 2010, 81,597 cotton farming households in these provinces had standing contractual agreements with Dunavant. Cotton is the primary cash crop for many small-scale farmers in the area. Most typically, small scale farms use one or two hectares for maize (as their primary source of nutrition) and about one hectare for cotton as their primary cash crop [15].

### Study design

**Figure 1:**
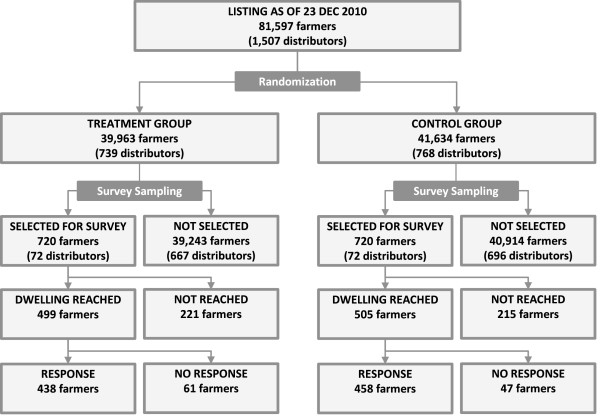
Study design.

The study used a cluster-randomized design as illustrated in Figure 1. The study population was composed of all 81,597 farmers who had a standing collaborative agreement with Dunavant Cotton for the 2010/11 season as of December 23^th^, 2010. The administrative database provided to the study by Dunavant contained commercial information on loans and farming inputs, but little information on households’ socioeconomic background.

### Randomization

ITNs were distributed following a stratified cluster-randomized design. A complete listing of farmers was extracted from Dunavant’s database on December 23^th^, 2010. In order to ensure a balanced rollout across regions, each of the 62 regional distribution offices (“sheds”) was treated as separate stratum in the randomization. Within each stratum, 49% of clusters were assigned to the ITN programme through a random number draw generated by Stata© 11 statistical software package. In most cases, clusters corresponded to all farmers in a given village working with Dunavant, though some of the larger villages had multiple distributors, which were then considered as separate clusters. Based on the available number of ITNs, 39,963 farmers were assigned to the treatment group, while 41,634 farmers were assigned to the control group.

### Study procedures

**Figure 2:**
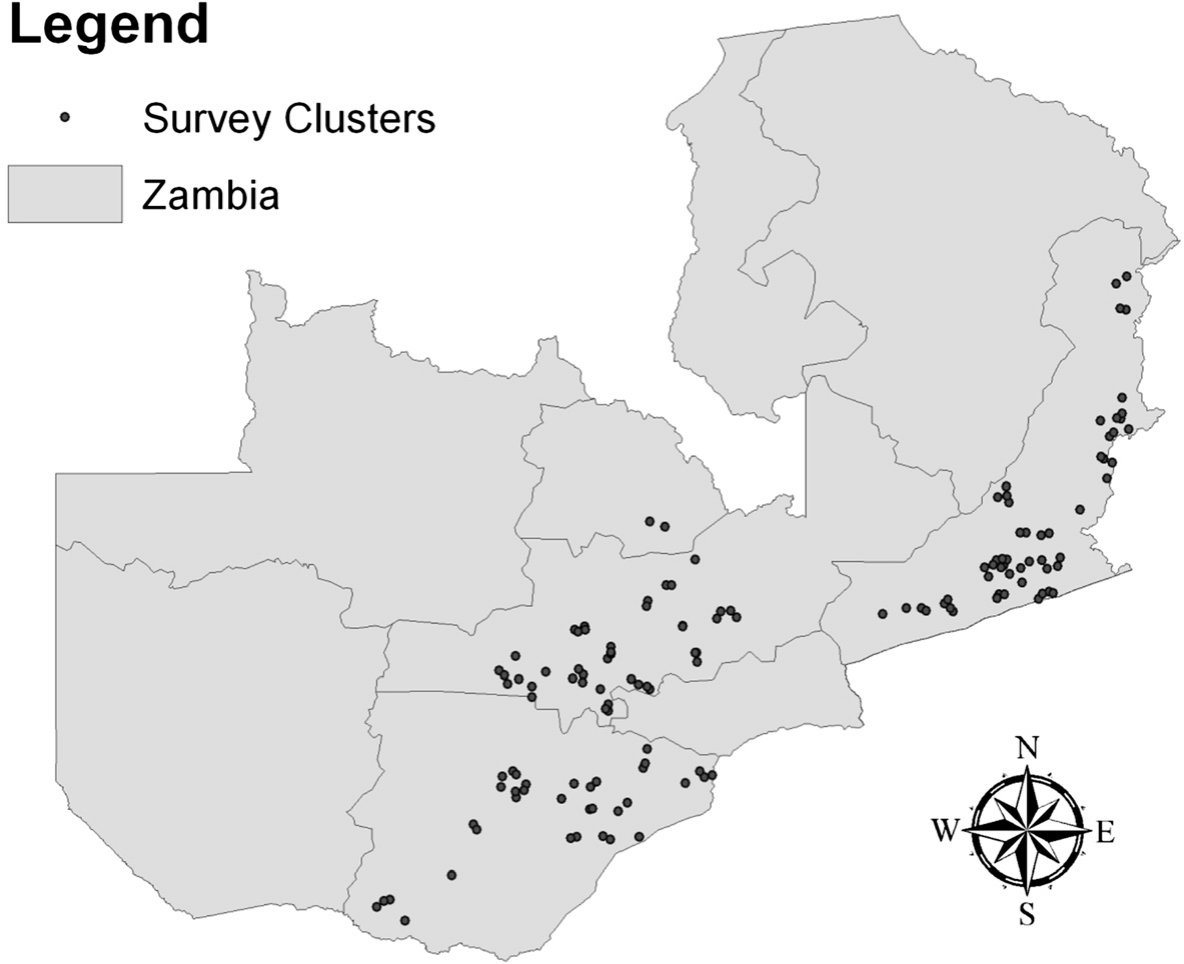
Spatial distribution of survey clusters.

Following the randomization, ITNs were distributed between January 20^th^ and January 28^th^ 2011. As a fair and simple distribution rule, it was determined that each household would receive exactly one ITN through the programme. ITNs were provided for free and without conditions, and could explicitly be kept even by farmers who would choose to discontinue the contractual relationship with Dunavant. In order to evaluate the impact of the distribution at the household level, and to verify the accuracy of the ITN distribution, a household survey was conducted between June 20^th^ and July 11^th^ in a randomly selected subset of 144 clusters. The household survey sampling followed a 3-step procedure. In a first step, 36 regional offices were randomly selected from the 62 offices operated by Dunavant. In a second step, two treatment and two control clusters were randomly selected, resulting in a total of 144 clusters as illustrated in Figure 2. In a last step, 10 farmers were randomly selected in each cluster. Within each cluster, surveyors were asked to find as many farmers as possible, with a minimum expected tracking rate of 70%. Across the 144 selected clusters, 1,004 of the 1,440 eligible respondent’s dwellings were identified. In 108 cases, informed consent could not be obtained, usually because of the absence of adults at the time of the survey. As a consequence, the final number of surveys collected was 896, with 438 respondents from the treatment group and 458 from the control group. All survey data was collected on paper and double-entered using the CSPro 4 software package.

### Outcome measures

#### ITN ownership and coverage

Household respondents were asked to list all individuals living on their plot, and then indicate for each of these whether or not they had been sleeping under an ITN the night before the interview. Bed net ownership was then verified by survey staff, who recorded the number of ITNs hanging in the dwellings.

#### Morbidity outcomes

In order to assess the health impact of the ITN distribution, household respondents were asked to indicate for each household member whether they had been sick with fever over the two-week period preceding the interview. Given Zambia’s sustained efforts to enforce comprehensive testing of all fever cases reporting at public health facilities, additional questions regarding the treatment of fevers were asked. For each fever case reported, respondents were asked whether a malaria blood test was performed; if the answer was affirmative, respondents were asked about the diagnostic result. To distinguish fever from presumed malaria cases, two separate indicator variables were analysed: a fever indicator, which was coded to one whenever a household member was reported to have suffered from a fever during the two weeks preceding the interview, and a presumed malaria indicator, which was coded to one if the responded reported that the fever case had been diagnosed as malaria.

Malaria incidence in Zambia fluctuates considerably over the course of a year (peaking in rainy season and abating in dry season), while the morbidity estimates presented here are derived from the two weeks prior to the interview. The survey period (June and July) corresponds to the end of the malaria transmission season, when the burden of malaria is very close to (if slightly below) the annual average. According to Zambia’s 2011 Health Management Information System, the months of June and July accounted for 8.2% and 5.9% of yearly malaria cases [16].

### Statistical analysis

Basic descriptive statistics were computed for bed net ownership and coverage in both the treatment and control groups. In order to quantify the relative risk reduction achieved by additional ITN distribution, standard logistic regression models were estimated using self-reported fever and reported confirmed malaria as dependent variables. To investigate whether the additional ITN particularly benefits children under five as the most vulnerable population group, separate models for the children under the age of five were estimated. To adjust for the spatial correlation of regression residuals, standard errors were clustered at the distributor level. All analysis was conducted using the Stata© 11 statistical software package.

## Results

### Baseline characteristics and balance test

Table 1 provides descriptive statistics at the household level by study arm. Average household size was 6.3, with nearly half of the household members being under the age of 15. Farms were rather small on average, using approximately two hectares to grow maize and one hectare to grow cotton on average, with much smaller amounts of lands used for other crops such as groundnuts, sun flowers and sweet potatoes. As Table 1 shows, no differences were detected between treatment and controls with respect to household composition or farm size.

**Table 1:** Household characteristics

	**Control**		**Intervention (Treatment)**		**Differences**^**a)**^	
	**N=458**		**N=438**			
	**Mean**	**Std.dev.**	**Mean**	**Std.dev.**	**Mean**	**p-value**
Children under 5	0.98	0.98	0.99	1.00	0.01	0.894
Children 5-14	2.04	1.72	1.98	1.73	−0.05	0.695
Ages 15 and older	3.47	1.86	3.29	1.79	−0.17	0.312
Maize area (hectares), 2010-11	1.87	1.95	1.79	2.09	−0.08	0.674
Cotton area (hectares), 2010-11	1.23	1.12	1.15	0.86	−0.08	0.445

a) Mean differences are adjusted for 144 clusters in the survey data.

### ITN receipts

As shown in Table 2, targeting appears to have been fairly accurate, with less than 5% of ITNs reported missing in the treatment group.

**Table 2:** “Did this household receive a mosquito net from Dunavant this season?”

	**Control**	**Intervention (Treatment)**
Yes	3 (0.7%)	413 (94.3%)
No	452 (98.7%)	20 (4.6%)
No response / don’t know	3 (0.7%)	5 (1.1%)
Total	458 (100%)	438 (100%)

### Impact on ITN utilization

**Figure 3:**
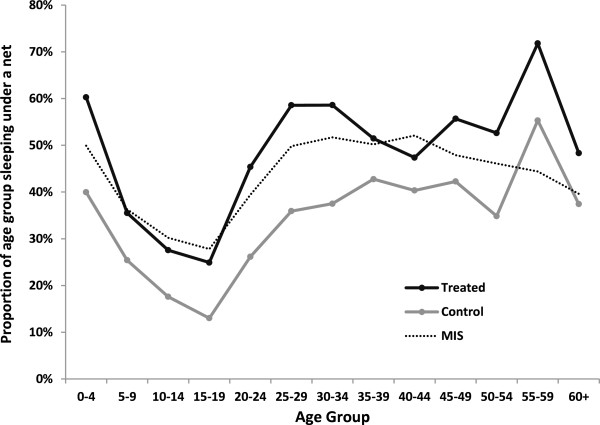
Self-reported net usage by treatment arm comparison to 2010 Malaria Indicator Survey (MIS).

Figure 3 shows average utilization rates in the treatment and control groups. In the control group, utilization of the available ITNs was approximately 40% for children under the age of five, and at comparable levels for adults 25 and older. The least protected group were children and teenagers between the ages of 5 and 19; on average, just over 10% of children of this age group were found sleeping under an ITN the night preceding the interview. These intra-household utilization patterns follow the NMCP’s emphasis on prioritizing protection for newborns and pregnant women. The impact of the net distribution is fairly consistent across all age groups: on average, age-specific utilization rates appear close to parallel, with a mean increase in the likelihood of ITN utilization of approximately 15 percentage points. Relative improvements are highest among teenagers between the ages of 15 and 19; net usage nearly doubled in this group, though in absolute terms, the age group remains the least protected.

### Impact on morbidity

**Figure 4:**
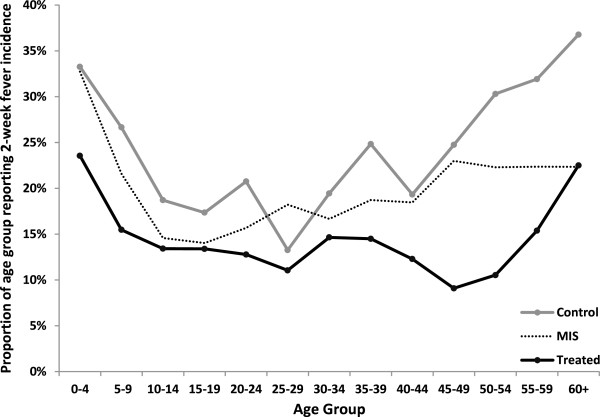
Self-reported fever incidence by treatment arm, comparison to 2010 Malaria Indicator Survey (MIS).

Table 3 provides an overview of self-reported morbidity outcomes. A total of 1,131 fever cases were reported, 528 of which were coded as presumed malaria. In the control group, the prevalence of self-reported fever was close to 24%, and the prevalence of presumed malaria was close to 12%. Figure 4 compares these data to the fever prevalence reported in Zambia’s 2010 Malaria Indicator Survey (MIS), which had been conducted one year earlier (in the months between April and May).

**Table 3:** Morbidity outcomes

	**Observations**	**Fever cases**	**Presumed malaria cases**
Control	2,968	707 (23.82%)	350 (11.79%)
Treated	2,744	424 (15.45%)	178 (6.49%)
Total	5,712	1,131 (19.80%)	528 (9.24%)

Table 4 shows multivariate results for the health impact of the additional ITN distributed through the programme. In columns 1–3 of Table 4, the dependent variable was self-reported fever during the two weeks preceding the interview. In columns 4–6, the dependent variable was presumed malaria. Columns 2 and 5 show the results for children under the age of five.

**Table 4:** Programme impact on morbidity

**Dependent**	**Fever last two weeks**			**Presumed malaria**		
	(1)	(2)	(3)	(4)	(5)	(6)
	OR	OR	OR	OR	OR	OR
Sample	All ages	Ages 0-4	Age 5+	All ages	Ages 0-4	Age 5+
N	5,712	881	4,831	5,712	881	4,831
Treated	0.580***	0.618**	0.570***	0.511***	0.653*	0.469***
(95% CI)	(0.427 - 0.788)	(0.413 - 0.926)	(0.413 - 0.787)	(0.335 - 0.782)	(0.401 - 1.064)	(0.297 - 0.741)

OR=Odds ratio . 95% CI are adjusted for clustered survey sampling. Estimates in columns 1,3,4 and 6 include 5-year-age-group dummies to control for age specific health risks.

*** p<0.01, ** p<0.05, * p<0.1.

On average, individuals in the treatment group faced 42% lower odds of suffering a fever, and 49% lower odds of reporting a confirmed case of malaria. The marginal impact appears to be slightly smaller for children under the age of five, which can likely be attributed to the higher average ITN coverage at baseline.

### Programme cost

ITNs (Brand: BASF Interceptor) were acquired at a wholesale price of $4.31. In addition, the NMCP paid $0.30 in direct costs to support the core distribution process. This number is substantially lower than benchmark distributions in sub-Saharan Africa [17,18] in part because core activities (such as the identification and sensitization of the target population as well as the storage, local distribution, and tracking of ITNs) were carried by Dunavant. Because of the organization’s existing infrastructure and organizational capacity in the distribution of farming inputs to the target population, these costs were relatively small, and, from a company perspective thought to be offset by the goodwill generated among partnering farmers. Compensation amounted to 12 US$ per treatment distributor, and 19 US$ for the shed managers who oversaw ITN distribution to the distributor level. Distributor payments were contingent on achieving at least 95% distribution accuracy according to Dunavant’s internal tracking systems. Table 5 provides a detailed breakdown of costs incurred by the NMCP. General and administrative costs cover administrative expenses associated with contracting issues. Factoring in a leakage rate of 4.6%, the total cost per successfully targeted ITN added up to $5.10. Some additional distribution costs were incurred because of unforeseen delays in the delivery of ITNs to Zambia: once the ITNs arrived, the NMCP opted to use its existing logistical infrastructure to distribute them to the shed level, rather than leaving the task to Dunavant as originally agreed. However, this was only done to adhere to the study schedule, which is why the associated economic costs of 0.49 US$ per ITN should be considered nonrecurring and, therefore, excluded. Also excluded are costs associated with study origination, design and analysis.

**Table 5:** Distribution costs to the NMCP

**Item**	**Campaign costs**	**per ITN**
ITNs	$172,240.53	$4.31
Supplies	$325.84	$0.01
Shed manager rewards	$1,158.35	$0.03
Distributor rewards	$5,959.09	$0.15
Logistics management	$4,464.00	$0.11
General and administrative	$10,400.00	$0.26
Total	$194,547.82	$4.87
Total, adjusted for leakage	$203,928.53	$5.10

Note: In accordance with WHO cost effectiveness standards [21], page 44 numbers are presented in international dollars, meaning that tradables (ITNs, supplies, logistics management) are converted into US$ using market exchange rates, while non-tradables (rewards) are converted using purchasing power parity-adjusted exchange rates.

## Discussion

The results presented in this paper are notable for a number of reasons. First, they show that engaging the private sector in distribution efforts can successfully improve ITN coverage in areas traditionally targeted by public campaigns. Second, the results show that there is both a need of, and an appreciation for, further ITNs, as highlighted by utilization improving across all ages in the intervention group relative to the control group. Third, and most importantly, the results suggest that additional ITNs had a significant impact on the self-reported fever and malaria. The estimated magnitude of the health effects appear large, though they are within the range of the estimates observed in controlled trial settings [7] as well as under other programmatic settings [19,20]. Further, the beneficial health effect was observed across age groups and particularly large among teenagers and adults, corresponding to the increase in utilization that was observed by the additional ITNs.

Overall, the distribution campaign analysed appears highly cost-effective under current WHO guidelines [21]. The data collected in this paper suggests an average of approximately 0.75 cases per household and two-week period in the absence of additional ITNs. Taking this rate as an annual average, this implies a total malaria burden of about three cases per person and year, or about 20 cases per household and year. The impact estimates presented in this paper suggest a reduction of 45% in the burden of malaria, which translates to a reduction of nine cases of malaria per household and year. The Roll Back Malaria Partnership currently assumes that 80% of distributed ITNs remain in use in the second year, and 50% in the third [22]. This implies that each ITN averts 24 malaria cases over the course of its life on average. With an estimated cost of $5.10 per ITN (and ignoring the time value of money), this translates into a $0.21 cost per malaria case averted. Assuming a case fatality rate of 3.8 per 1,000 as suggested in the 2008 WHO country profile [23], this translates into a cost of approximately $55 to the NMCP per malaria death averted. Under a more conservative case fatality rate of one per 1,000 as suggested by health facility records in the Zambian Health Management Information System database, the estimated cost per death averted would be US$ 210. Under both scenarios, continued distribution of ITNs in this manner should be considered highly cost-effective, following current guidelines [24]. This may be attributed to the high baseline incidence of malaria, high ITN usage rates, as well as low distribution cost. To provide a benchmark, the WHO estimates the cost per death averted to be US$ 212 on the optimistic assumptions that ITNs can be exclusively targeted to the age group with highest mortality rates (children under 5) and have a 3 year lifespan [25].

This study has several limitations. The first and likely most important limitation of the study lies in its reliance on self-reported morbidity outcomes. Recent studies in Uganda [26] as well as Zambia (Eisele at al, 2012, personal communication) suggest that there are challenges with accurate recall of malaria diagnoses at household level. It is possible that survey respondents may have over-reported incidences of fever and malaria; meanwhile, given that only a fraction of fever cases got tested for malaria, the morbidity variable may underestimate the true disease burden. For analytical purposes, the main interest of the study lies in the relative risk reduction, which is not directly affected by these biases as long as these are not affected by the treatment itself.

From a cost-effectiveness perspective, a second limitation of the study is the lack of reliable mortality data. While case fatality rates from other studies can be applied to the collected data, actual rates may differ and display substantial regional variations due to differential access to proper diagnosis and treatment.

Lastly, the study design is applicable only to restricted settings because the capacity of Dunavant as a distribution channel is limited: as of 2012, the company works with approximately 140,000 contract farming households. While the entire Zambian cotton industry may work with as many as 300,000 households (close to two million individuals), this still accounts for less than 20% of Zambia’s population. Only a fraction (which varies substantially across villages) of households in each location is actively engaged in contract farming networks, and it is not clear if these could effectively reach individuals that they do not have pre-existing contractual commitments with. Further research could address this issue.

## Conclusions

The challenge of sustaining coverage levels represents an ongoing struggle for malaria control programmes in Africa. This study illustrates that private sector companies can be cost-effectively integrated into large-scale ITN distribution campaigns.

### IRB Approval

All study procedures were approved by the University of Zambia Research Ethics Committee and the PATH Research Ethics Committee (study file number: HS 564).

## Competing interests

The authors declare that they have no competing interest.

## Authors’ contributions

RS designed and implemented the study. GF supervised the randomization and conducted the initial empirical analysis. JM assisted in the design and implementation of the study. All authors contributed to the drafting and editing of the manuscript.
